# Breakthroughs in modern brachytherapy are transforming cancer care^☆^

**DOI:** 10.1016/j.phro.2025.100783

**Published:** 2025-05-17

**Authors:** Georgina Fröhlich, Alfonso Gomez-Iturriaga, Gemma Eminowicz, Christoph Bert, Åsa Carlsson Tedgren, Alexandra J. Stewart, Bernd Wisgrill, Ludvig P. Muren

**Affiliations:** National Institute of Oncology, Centre of Radiotherapy, Budapest, Hungary; Eötvös Loránd University, Faculty of Sciences, Budapest, Hungary; Cruces University Hospital, Biobizkaia Health Research Institute, Basque Country University (UPV/EHU), Spain; University College London Hospital, London, UK; Department of Radiation Oncology, Universitätsklinikum Erlangen, Erlangen, Germany; Karolinska University Hospital, Stockholm, Sweden; Linköping University, Stockholm, Sweden; Royal Surrey Hospital, Guildford, UK; University of Surrey, Guildford, UK; Department of Radiation Oncology, Universitätsklinikum AKH Wien, Vienna, Austria; Danish Centre for Particle Therapy, Aarhus University Hospital, Aarhus, Denmark; Department of Clinical Medicine, Aarhus University, Aarhus, Denmark

Brachytherapy is a cornerstone of modern radiotherapy that continues to evolve through the dynamic interplay of clinical innovation and technological advancement. Decision-making is heavily influenced by time pressure during the whole procedure, fostering a uniquely close collaboration and dynamic interplay among radiation oncologists, medical physicists, radiation therapists, and other professionals, forming an interdisciplinary foundation that is essential for both innovation and quality assurance [1]. Reflecting this interdisciplinary momentum, all three open-access journals of ESTRO have been collecting and publishing brachytherapy-related papers in the first-ever joint virtual special issue across the journals. This editorial is written to show how these papers are contributing to the further strengthening of this evolving field.

The publications included in the special issue cover many areas, from artificial intelligence to 3D printing, and from IAEA portal dosimetry audits to patient-reported outcomes, addressing challenges relevant across multiple tumour sites. Overall, the papers show joint efforts across specialities, catalysing future development and strengthening the role of brachytherapy within the broader radiotherapy landscape. This synergy is particularly evident in the findings of the new Brachy-HERO study by Johansen et al. [2], which underscores the need for harmonized practices and multidisciplinary engagement to optimize outcomes. This should be seen in the light of the expected increase in the number of radiotherapy treatment courses, e.g. a previous HERO study predicted a 16 % increase from 2012 to 2025 [3]. The effect of brachytherapy is important not only at the global level but also at the level of individual patients. Spyrou et al. [4] highlighted the essential role of integrating patient-reported outcomes to assess the real-world impact of brachytherapy on quality of life. They emphasise standardised tools, timing, and digital integration for meaningful data collection. Building on this patient-centred perspective, recent advances in radiobiological modelling offer deeper insight into how biological mechanisms at cellular and tissue levels can inform and refine these clinical observations [5]. A review about the radiobiological aspects of brachytherapy explores the complex interplay of dose distribution, radiobiological modelling, and clinical response, stressing the need for individualised treatment plans and advanced modelling to optimise therapeutic outcomes [6].

The mentioned Brachy-HERO survey also highlights the widespread use of brachytherapy for gynaecological cancers currently, as well as for the future [2]. However, the delivery of image-guided adaptive brachytherapy can be resource-intensive as well as time-consuming [7]. With that in mind, efforts to streamline or speed up workflows are ongoing. In line with published reviews highlighting clinical workflow efficiency improvements through the use of artificial intelligence [8,9], Fionda et al. [10] and Simoes et al. [11] demonstrated time-saving benefits of auto-segmentation and automated planning processes, respectively, without compromising clinical quality. Dohlmar et al. [12] applied pseudo-structures to successfully shape isodoses and lowered loading in needles, yet another method to use automation to ease the implementation of interstitial brachytherapy. Shifting focus from artificial intelligence, service efficiencies in vaginal vault brachytherapy delivery with the introduction of an advanced practice radiation therapist were reported by Goodwin [13] consistent with a previous Canadian pilot study [14]. Alongside efficiency, the accuracy of treatment is increasingly important due to the tailoring of dose prescriptions and the increasing use of interstitial needles [15]. Accuracy of needle placement is key, and Cantin et al. [16] demonstrated the feasibility of an electromagnetic tracking system to guide needle placement, which could be investigated further in clinical practice. Importantly, the verification of the actual treatment delivered using *in vivo* dosimetry was the focus of two articles, which highlighted the lack of standardisation of *in vivo* dosimetry in gynaecological brachytherapy despite the knowledge that small changes could lead to large shifts in delivered doses. An original article by Georgi et al. [17] and a literature review by Chowdhury et al. [18] both showed the feasibility of *in vivo* dosimetry while acknowledging this is not yet integrated into clinical practice. In addition to *in vivo* dosimetry, modern imaging modalities like MRI can also make a valuable contribution to treatment verification. Van Vliet-van den Ende et al. [19] presented the clinical benefits of their workflow using a combined 1.5 T magnetic resonance imaging/high-dose-rate brachytherapy suite for adaptive brachytherapy of cervical cancer. They show that repeated magnetic resonance imaging before and/or after irradiation allowed individual anatomical changes to be taken into account and applicator and needle positions to be adapted if indicated. This enabled a more personalised therapy and improved the estimation of the actual dose delivered. To add perspective to these discussions of increasing technology and automation, it is important to understand that countries and regions with the highest cervical cancer incidence (e.g., in Africa), have inadequate access to brachytherapy as described by Fiagbedzi et al. [20]. Increasing access to this modality in these countries is likely to have a far greater impact on clinical outcomes than improvements in workflow efficiency and accuracy.

Building on this, recent developments in urological brachytherapy further emphasise the crucial role of advanced technology and multidisciplinary collaboration. One of the most notable advancements in recent years in prostate cancer radiotherapy is dose escalation to regions with higher tumour burden within the prostate, specifically targeting dominant intraprostatic lesions, while minimising side effects to surrounding organs at risk [21]. This strategy demands an exceptionally high level of precision. One way of achieving this goal is the implementation of deep learning models for automatic reconstruction of high-dose-rate interstitial needles in prostate cancer, as demonstrated by Moradi et al. [22], exemplifying how artificial intelligence can streamline workflows, reduce human error, and significantly enhance spatial accuracy. In line with this, the review by Poder et al. [23] identified clinical and planning studies that demonstrated that brachytherapy provides an effective method for dose escalation, with, in most cases, acceptable or reduced toxicity compared to external beam radiotherapy, and highlighted the dose deposition characteristics of brachytherapy as an attractive option for focal dose escalation. In addition to high-dose-rate applications, low-dose-rate brachytherapy using iodine-125 sources for prostate seed implants is also a well-established treatment option for patients with localised prostate cancer. Sen et al. [24] described improvements in the workflow of adaptive low-dose-rate brachytherapy that could also be possible with the help of modern software applications. Precision-driven approaches depend critically on close collaboration between medical physicists, radiation oncologists, radiologists, and engineers. These improvements in accuracy and personalisation have clear potential to translate into better outcomes. It is well established that incorporating patient-reported outcomes into cancer treatment has the potential to enhance overall survival and health-related quality of life [25]. In the case of prostate cancer, where curative therapies are typically highly successful and long-term survival is common, preserving quality of life becomes crucial. In this context, Spyrou et al. [4] emphasised the critical importance of using patient-reported outcomes to evaluate the real-world effects of brachytherapy on patients' quality of life. Therefore, combining advanced artificial intelligence driven planning with focal treatment strategies holds the promise not only of improved cancer control but also of preserving the well-being of patients. In this regard, prostate brachytherapy emerges as a pivotal modality capable of integrating high-precision delivery, individualised care, and sustained quality of life in modern prostate cancer management [26].

While prostate and gynaecological treatments represent the majority of clinical applications of brachytherapy, the modality is also very successful in other treatment sites. Among such sites are treatments of breast tumours for which Pignier et al. [27] investigated whether the removal of the implant can safely be done by radiation therapists to make the clinical workflow more efficient. They gradually and successfully trained this group of the medical team over 15 years, during which >400 patients were treated. The paper by Sölkner et al. [28] reported on the few adverse events observed clinically, which could be collected and potentially mitigated by dedicated reporting and risk management systems. Optimisation of treatment is also conducted concerning dose calculation algorithms. AAPM TG-43 is still the standard despite its simplifications and the availability of algorithms according to TG-186. As Faucher et al. [29] reported, widespread clinical implementation of model-based dose calculation algorithms will require data that correlates clinical outcomes to the computed doses of these algorithms. Their paper helps with that goal by comparing dose calculations for the different algorithms and dose to water/media in breast cancer settings. In support of advancing brachytherapy practices, the study by Rossi et al. [30] contributed valuable insight by comparing dose calculations across different algorithms and assessing dose-to-water versus dose-to-media in breast cancer settings. Extending this comparative approach, they also evaluated model-based dose calculations for extensive scalp lesions, concluding that such methods could enable adaptive brachytherapy guided by daily cone-beam computed tomography imaging. Although superficial brachytherapy remains a relatively rare modality, several contributions are addressing this application. In the realm of medical physics, Szlag et al. [31] explored various image registration techniques relevant to melanoma treatment, a context in which dose accumulation from sequential sessions is often necessary. While such technological advancements are essential to refining treatment delivery, clinical outcomes ultimately remain the benchmark for therapeutic success. Addressing this, Budrukkar et al. [32] presented data on local control, toxicity, functional outcomes, and cosmetic results in patients with non-melanoma skin cancers. A range of therapeutic options exists for the management of these lesions; however, recent years have seen the introduction of 3D-printed applicators. These innovations offer the potential for highly individualized treatments by enabling the applicator design to be tailored precisely to each patient’s anatomy. An illustrative example of this personalised strategy was provided by De Ridder et al. [33], who described its application in the treatment of nasal vestibule cancer using pulsed dose rate brachytherapy, thereby reducing the necessity for interstitial catheters.

Whether applied to breast tumours, scalp lesions, or anatomically complex and superficial sites, the growing versatility of brachytherapy across diverse indications reinforces the central role of advanced dosimetry − not only as a technical necessity but also as a driving force behind increasingly individualised, precise, and effective care [34]. The publications about dosimetry and technical aspects underscore major advancements in brachytherapy – from improving dosimetric accuracy and inter-institutional quality control via postal audits, to leveraging artificial intelligence for more responsive, patient-centred treatment. They collectively highlight the potential for technology and collaboration to elevate both technical precision and holistic patient care in modern brachytherapy. Dimitriadis et al. [35] presented a novel, standardised postal audit system to verify dosimetric accuracy across high-dose rate brachytherapy centres, promoting global quality assurance and consistency. Complementing this, the group of Chelminski [36] addressed dosimetric uncertainties by providing refined Monte Carlo-based correction factors, improving the precision and reliability of the postal audit measurements. Meanwhile, Fionda et al. [10] explored how artificial intelligence integration can transform brachytherapy workflows, from treatment planning to follow-up, while also personalising care through predictive analytics and automation, ultimately enhancing patient experience and outcomes.

As these technical and digital innovations continue to refine the precision and personalisation of brachytherapy, they also lay the groundwork for its integration with emerging treatment paradigms, most notably, immunotherapy, where the biological impact and spatial dose distribution of brachytherapy open exciting new therapeutic frontiers [37]. As highlighted by Serre et al. [38], the unique dose heterogeneity of brachytherapy can modulate the tumour microenvironment to enhance immune responses. High-dose regions may induce immunogenic cell death and antigen release, while lower doses can stimulate immune cell infiltration and cytokine production. This spatial dose variation positions brachytherapy as a promising partner for immunotherapies, potentially transforming tumours into in situ vaccines [39]. Preclinical studies have demonstrated that combining brachytherapy with immune checkpoint inhibitors can lead to systemic anti-tumour effects, including responses in non-irradiated lesions, known as the abscopal effect [40]. Realising these benefits necessitates close collaboration among radiotherapy professionals and immunologists. Brachytherapy stands at a pivotal intersection of technological sophistication, clinical versatility, and biological innovation. From refined dosimetry and artificial intelligence driven workflows to its potential synergy with immunotherapy, the field is rapidly evolving toward a more personalised, interdisciplinary model of care. This transformation is not only expanding the boundaries of what brachytherapy can achieve but also reinforcing its role as a cornerstone of modern oncologic treatment.

## Declaration of competing interest

The authors declare that they have no known competing financial interests or personal relationships that could have appeared to influence the work reported in this paper.

Georgina Fröhlich is an Editorial Board Member and Ludvig Muren is an Editor-in-Chief for *Physics and Imaging in Radiation Oncology* and were not involved in the editorial review or the decision to publish this article.

## References

[1] Zoberi J.E., Meyer J., Al-Hallaq H.A., Landers A., Leif J., Kim H. (2024). Distribution of responsibility across the HDR brachytherapy team: results of a nationwide survey. Int J Radiat Oncol Biol Phys.

[2] Johansen J.G., Jürgenliemk-Schulz I.M., Haddad H., Hannoun-Levi J.M., Hellebust T.P., Guix B. (2024). Current status of brachytherapy in Europe. Clin Transl Radiat Oncol.

[3] Borras J.M., Lievens Y., Barton M., Corral J., Ferlay J., Bray F. (2016). How many new cancer patients in Europe will require radiotherapy by 2025?. An ESTRO-HERO analysis Radiother Oncol.

[4] Spyrou A., Martin A.-G., Hannoun-Lévi J.-M., Stewart A. (2024). Measuring patient-reported outcomes in brachytherapy: why we should do it and more importantly how. Clin Transl Radiat Oncol.

[5] Afshari N., Koturbash I., Boerma M., Newhauser W., Kratz M., Willey J. (2024). A review of numerical models of radiation injury and repair considering subcellular targets and the extracellular microenvironment. Int J Mol Sci.

[6] Stewart A.J., Chargari C., Chyrek A., Eckert F., Guinot J.L., Hellebust T.P. (2024). Radiobiology and modelling in brachytherapy: a review inspired by the ESTRO Brachytherapy pre-meeting course. Clin Transl Radiat Oncol.

[7] Helley C.E., Barraclough L.H., Nelder C.L., Otter S.J., Stewart A.J. (2021). Adaptive radiotherapy in the management of cervical cancer: review of strategies and clinical implementation. Clin Oncol (R Coll Radiol).

[8] Liu T., Wen S., Wang S., Yang Q., Wang X. (2024). Artificial intelligence in brachytherapy. J Radiat Res Appl Sci.

[9] Chen J., Qui R.L.J., Wang T., Momin S., Yang X. (2025). A review of artificial intelligence in brachytherapy. J Appl Clin Med Phys.

[10] Fionda B., Placidi E., de Ridder M., Strigari L., Patarnello S., Tanderup K. (2024). Artificial intelligence in interventional radiotherapy (brachytherapy): enhancing patient-centered care and addressing patients' needs. Clin Transl Radiat Oncol.

[11] Simões R., Rijkmans E.C., Schaake E.E., Nowee M.E., van der Velden S., Janssen T. (2024). Evaluation of deep learning-based target auto-segmentation for Magnetic Resonance Imaging-guided cervix brachytherapy. Phys Imaging Radiat Oncol.

[12] Dohlmar F., Morén B., Sandborg M., Larsson T., Carlsson T.Å. (2024). Dwell time shaping in inverse treatment planning for cervical brachytherapy. Phys Imaging Radiat Oncol.

[13] Goodwin R. (2024). Advanced practice radiation therapist led vaginal vault brachytherapy: an evaluation of efficiency and effectiveness of service delivery. Tech Innov patient Support Radiat Oncol.

[14] Harnett N., Bak K., Lockhart E., Ang M., Zychla L., Gutierrez E. (2018). The clinical Specialist Radiation Therapist (CSRT): a case study exploring the effectiveness of a new advanced practice role in Canada. J Med Radiat Sci.

[15] Ladbury C., Harkenrider M., Taunk N., Fisher C., Mayadev J., Venkat P. (2023). A practical guide to hybrid interstitial/intracavitary brachytherapy for locally-advanced cervical cancer. Brachytherapy.

[16] Cantin A., Lavallée M.-C., Poulin E., Hautvast G., Foster W., Beaulieu L. (2024). Proof-of-concept of real-time electromagnetic guidance for gynecologic interstitial catheters in high dose rate brachytherapy. Phys Imaging Radiat Oncol.

[17] Georgi P.D., Nielsen S.K., Hansen A.T., Spejlborg H., Rylander S., Lindegaard J. (2024). In vivo dosimetry with an inorganic scintillation detector during multi-channel vaginal cylinder pulsed dose-rate brachytherapy: dosimetry for pulsed dose-rate brachytherapy. Phys Imaging Radiat Oncol.

[18] Chowdhury A.A., Bolton S., Lowe G., Vasquez Osorio E., Hamblyn W., Hoskin P.J. (2024). The clinical application of in vivo dosimetry for gynaecological brachytherapy: a scoping review. Tech Innov Patient Support Radiat Oncol.

[19] Vliet-van V., den Ende K.M., Hoogendoorn-Mulder P.G., Schokker R.I., Moerland M.A., Kroon P.S. (2024). Adaptive brachytherapy for cervical cancer in combined 1.5 T MR/HDR suite: impact of repeated imaging. Tech Innov Patient Support Radiat Oncol.

[20] Fiagbedzi E., Atuwo-Ampoh V.D., Ofori I.N., Nyarko S., Adomako A., Hasford F. (2024). Access to cervical cancer brachytherapy in Africa. Clin Transl Radiat Oncol.

[21] Kerkmeijer L.G.W., Groen V.H., Pos F.J., Haustermans K., Monninkhof E.M., Smeenk R.J. (2021). Focal boost to the intraprostatic tumor in external beam radiotherapy for patients with localized prostate cancer: results from the FLAME randomized phase III trial. J Clin Oncol.

[22] Moradi M.M., Siavashpour Z., Takhtardeshir S., Showkatian E., Jaberi R., Ghaderi R. (2025). Fully automatic reconstruction of prostate high-dose-rate brachytherapy interstitial needles using two-phase deep learning-based segmentation and object tracking algorithms. Clin Transl Radiat Oncol.

[23] Poder J., Hoskin P., Reynolds H., Chan T.H., Haworth A. (2024). A review of whole gland prostate brachytherapy with focal dose escalation to intra-prostatic lesions: clinical efficacy and technical aspects. Phys Imaging Radiat Oncol.

[24] Sen S., Stolen E., Chun J., Sung Kim J., Sohn J.J. (2024). Optimized needle configuration for operational seed (ONCOSEED) efficiency and deployment for prostate seed implants. Tech Innov Patient Support Radiat Oncol.

[25] Balitsky A.K., Rayner D., Britto J., Lionel A.C., Ginsberg L., Cho W. (2024). Patient-reported outcome measures in cancer care: an updated systematic review and meta-analysis. JAMA Netw Open.

[26] Prashar J., Schartau P., Murray E. (2022). Supportive care needs of men with prostate cancer: a systematic review update. Eur J Cancer Care (Engl).

[27] Pignier M., Rene L., Carenco J., Dubosc M., Moreau M., Rizzi Y. (2024). Catheter removal after breast BT: task delegation. Tech Innov Patient Support Radiat Oncol.

[28] Sölkner L, Georg D, Wolff U, Renner A, Widder J, Heilemann G. Enhancing clinical safety in radiation oncology: a data-driven approach to risk management. Z Med Phys 2025, in press. 10.1016/j.zemedi.2025.02.003. PMC12766486 40059000

[29] Faucher J., Turgeon V., Bahoric B., Enger S.A., Watson P.G.F. (2025). Isolating the impact of tissue heterogeneities in high dose rate brachytherapy treatment of the breast. Phys Imaging Radiat Oncol.

[30] Rossi G., Peppa V., Gainey M., Kollefrath M., Sprave T., Papagiannis P. (2024). On the impact of improved dose calculation accuracy in clinical treatment planning for superficial high-dose-rate brachytherapy of extensive scalp lesions. Phys Imaging Radiat Oncol.

[31] Szlag M., Stankiewicz M., Kellas-Ślęczka S., Stąpór-Fudzińska M., Cholewka A., Pruefer A. (2024). Comparison of image registration methods in patients with non-melanoma skin cancer treated with superficial brachytherapy. Phys Imaging Radiat Oncol.

[32] Budrukkar A., Singh M., Swain M., Ghosh Laskar S., Murthy V., Kale S. (2024). Interstitial brachytherapy for periocular nonmelanoma skin cancers: impact on organ and function preservation. Tech Innov Patient Support Radiat Oncol.

[33] de Ridder M., Smolic M., Kastelijns M., Kloosterman S., van der Vegt S., Rijken J.A. (2024). Individualized 3D-printed applicators for magnetic resonance imaging-guided brachytherapy in nasal vestibule cancer. Phys Imaging Radiat Oncol.

[34] Chargari C., Deutsch E., Blanchard P., Gouy S., Martelli H., Florent G. (2019). Brachytherapy: an overview for clinicians. CA Cancer J Clin.

[35] Dimitriadis A., Becker A., Chelminski K., Kazantsev P., Titovich E., Azangwe G. (2024). Development and international multicentre pilot testing of a postal dosimetry audit methodology for high dose rate brachytherapy. Phys Imaging Radiat Oncol.

[36] Chelminski K., Dimitriadis A., Abdulrahim R., Kazantsev P., Granizo-Roman E., Kalinowski J. (2024). Monte Carlo simulated correction factors for high dose rate brachytherapy postal dosimetry audit methodology. Phys Imaging Radiat Oncol.

[37] Mondini M., Levy A., Meziani L., Milliat F., Deutsch E. (2020). Radiotherapy–immunotherapy combinations – perspectives and challenges. Mol Oncol.

[38] Serre R., Gabro A., Andraud M., Simon J.-M., Spano J.-P., Maingon P. (2025). Brachytherapy: perspectives for combined treatments with immunotherapy. Clin Transl Radiat Oncol.

[39] Bo Y., Wang H. (2024). Biomaterial-based in situ cancer vaccines. Adv Mater.

[40] Nabrinsky E., Macklis J., Bitran J. (2022). A review of the Abscopal effect in the era of immunotherapy. Cureus.

